# Totally biological composite aortic stentless valved conduit for aortic root replacement: 10-year experience

**DOI:** 10.1186/1749-8090-6-86

**Published:** 2011-06-23

**Authors:** Manuel Galiñanes, Ayo Meduoye, Ignacio Ferreira, Andrzej Sosnowski

**Affiliations:** 1 Department of Cardiac Surgery, The Glenfield Hospital, Leicester, UK; 2 Department of Cardiac Surgery, Research Institute, University Hospital Vall d'Hebron, Universitat Autònoma de Barcelona, Barcelona, Spain; 3 Department of Cardiology, Reparative Therapy of the Heart, Area del Cor (ACOR) and Research Institute, University Hospital Vall d'Hebron, Universitat Autònoma de Barcelona, Barcelona, Spain

**Keywords:** aorta and aortic valve disease, aortic valved conduit, aortic root replacement, Bentall's operation, BioConduit, detoxified tissue, homografts, autografts

## Abstract

**Objectives:**

To retrospectively analyze the clinical outcome of a totally biological composite stentless aortic valved conduit (No-React^® ^BioConduit) implanted using the Bentall procedure over ten years in a single centre.

**Methods:**

Between 27/10/99 and 19/01/08, the No-React^® ^BioConduit composite graft was implanted in 67 patients. Data on these patients were collected from the in-hospital database, from patient notes and from questionnaires. A cohort of patients had 2D-echocardiogram with an average of 4.3 ± 0.45 years post-operatively to evaluate valve function, calcification, and the diameter of the conduit.

**Results:**

Implantation in 67 patients represented a follow-up of 371.3 patient-year. Males were 60% of the operated population, with a mean age of 67.9 ± 1.3 years (range 34.1-83.8 years), 21 of them below the age of 65. After a mean follow-up of 7.1 ± 0.3 years (range of 2.2-10.5 years), more than 50% of the survivors were in NYHA I/II and more than 60% of the survivors were angina-free (CCS 0). The overall 10-year survival following replacement of the aortic valve and root was 51%. During this period, 88% of patients were free from valved-conduit related complications leading to mortality. Post-operative echocardiography studies showed no evidence of stenosis, dilatation, calcification or thrombosis. Importantly, during the 10-year follow-up period no failures of the valved conduit were reported, suggesting that the tissue of the conduit does not structurally change (histology of one explant showed normal cusp and conduit).

**Conclusions:**

The No-React^® ^BioConduit composite stentless aortic valved conduit provides excellent long-term clinical results for aortic root replacement with few prosthesis-related complications in the first post-operative decade.

## Introduction

The aortic root replacement by the Bentall's procedure, described in 1968 [1], has been refined over time [2-4] and still represents the preferred treatment for patients with ascending aortic aneurysm and aortic valve disease in whom the David's or Yacoub's operation cannot be performed. Initially, the use of composite grafts with mostly mechanical valves was considered a good treatment, however the 10-year results are less than desirable [2,5]. This is mostly because of the complications of anticoagulation and low, but consistent, rates of infection that require removal of the Dacron graft, which carries a high rate of mortality [4,6]. In the presence of infection, replacement of the synthetic graft with a biological conduit is needed [6,7]. Therefore, there is a need to determine the "ideal" valved conduit, preferably totally biological, not requiring anticoagulation, and durable for all patient ages.

Among the biological conduits, allografts (also known as homografts) and pulmonary autografts (Ross procedure) have been considered for aortic root replacement, but the former are not always available and the latter is not always possible. However, short-term follow-up studies have clearly shown that, when compared to the composite mechanical valve conduits, allografts and pulmonary autografts have advantages only early after implantation [5], because allografts seems to have age-related limited durability [8] and after eight years follow-up the pulmonary autografts' freedom from moderate or severe regurgitation is below 75%, and freedom from dilatation is between 10-15% [9]. Furthermore, Pasquali *et al*. have shown that pulmonary autografts in the aortic position dilate for up to 60% of patients at 6 years follow-up [10]. Such a high rate of dilatation would lead to progressive rates of aortic valve dysfunction, a process that appears to start only after 3 years [9-11]. The causes of these detrimental changes are not fully elucidated but Schoof *et al*. [11] performing histological analysis on explanted pulmonary autografts from the Ross procedure demonstrated that the elastic tissue of the autograft had been slowly substituted with fibrous material, including both the conduit wall and the valve cusps, a "degeneration" considered as a negative "remodelling" process. It is clear that biological valved conduits alternative to allografts and pulmonary autografts are much needed, having in mind that the "ideal" bio-conduit should require no anticoagulation, can be implanted in patients of all ages, and should have no structural changes over the years. In addition, the ideal conduit should resist infection.

The No-React^® ^BioConduit is a valved conduit made from bovine pericardium made with the aim to resist foreign body reaction and degeneration without needing anticoagulation. Previous experimental animal studies have shown that No-React^® ^tissue causes no foreign body reaction, leading to a resistance to calcification and degeneration [12]. Further studies using No-React^® ^tissue as a patch for the Norwood procedure caused no anti-HLA antibodies whereas allografts induced the production of antibodies [13]. Indeed, clinical studies with No-React^® ^valves, receiving an identical treatment to the No-React^® ^BioConduit, have shown a high resistance to infection [14,15]. However, a recent report has warned on the possibility that this valved conduit may undergo degeneration [16]. For a number of years, the No-React^® ^BioConduit has been used in our institution in patients with low life expectancy and advanced disease of the aortic valve and root in whom the implantation of other valved conduits was not advisable or possible. Therefore, in this study, we have investigated the long-term clinical results with the Bentall procedure using the No-React^® ^BioConduit (BioIntegral Surgical, Inc., Canada, formerly manufactured by Shelhigh) over a 10-year period in high risk patients in a single centre.

### Patients and Methods

Between October 1999 and January 2008, 67 patients with significant aortic valve and root pathology received a No-React^® ^BioConduit. The preoperative characteristics of the study population are shown in Table 1. Of them, 40 were males (60%) and 27 females (40%) with a mean age of 67.9 ± 1.3 years (range 34.1-83.8 years), with 21 being below the age of 65. This also shows that approximately 40% of the patients suffered from angina and 80% were in NYHA class ≥ 2. Importantly, approximately 40% were or had previously experienced congestive heart failure with 20% of them being in a critical preoperative state. The advanced cardiac disease presented at the time of surgery was reflected by a high logistic EuroSCORE with a mean of 46.8.

**Table 1:** Patients' characteristics

	N valid	
*Age at surgery (mean; SD)*	67	67.9 (10) Range: 34-84

*Females*	67	27 (40.3%)

*BMI (mean; SD)*	62	26.2 (4.6) Range: 14-38

**Associated conditions**		

DM	67	1 (1.5%)

Hypertension	67	34 (50.7%)

Smoking status	67	

Never		29 (43.3%)

Ex -smoker		31 (46.3%)

Current		7 (10.4%)

Renal dysfunction	67	3 (4.5%)

Chronic Pulmonary Disease	67	11 (16.4%)

Cerebrovascular disease	67	5 (7.5%)

Peripheral vascular disease	67	4 (6%)

Previous Q wave MI	67	2 (3%)

**Preoperative clinical status**		

*Angina status (CCS class)*	67	

0		39 (58.2%)

1		8 (11.9%)

2		11 (16.4%)

3		9 (13.4%)

4		0

*Dyspnea status (NYHA class)*	67	

1		14 (19.9%)

2		23 (34.3%)

3		23 (34.3%)

4		7 (10.4%)

*Congestive heart failure*	67	

Never		42 (62.7%)

Now		18 (26.9%)

Past		7 (10.4%)

*Neurological dysfunction*	67	2 (3%)

*Preop arrythmias (AF/Flutter)*	67	8 (11.9)

*Left ventricular ejection fraction*	67	

> 50%		52 (77.6%)

30-50%		9 (13.4%)

< 30%		6 (9%)

*Critical preoperative state*	67	13 (19.4%)

*Preoperative pacemaker*	67	2 (3%)

*Extent of coronary disease*	67	

Grossly normal		55 (82.1%)

Single vessel		5 (7.5%)

Double vessel		4 (6%)

Triple vessel		3 (4.5%)

Left main stem disease		2 (3%)

*Logistic EuroSCORE (mean;SD)*	67	46.8 (19.3)

*Logistic EuroSCORE (P25, P50, P75)*	67	31.2; 45.3; 58.8

### Surgical Technique

Surgical procedures were performed under standard anesthetic protocol, operative techniques and post-operative care. Briefly, patients were given Temazepam 20 mg and ranitidine 150 mg as premedication 2 hours before their scheduled operation. Intravenous access was established in the induction room, before the patients were preoxygenated and monitored with ECG, pulse oximetry and arterial line pressure tracing. Anesthesia was then induced with fentanyl 5-10 μg/kg, midazolam 0.05-0.1 mg/kg and rocuronium 1 mg/kg, and maintained with O_2_/air mixture and isoflurane to achieve a Bispectral Index System reading of less than 50. Patients were then intubated and a central venous catheter was inserted. All operations were performed through a median sternotomy using standard techniques with cardiopulmonary bypass (CPB) under full heparinization (3-4 mg/kg intravenously), and regular doses of cold blood cardioplegia (ratio of blood to St. Thomas' cardioplegic solution No1 of 4:1. 1000 ml was given during the first dose, subsequently 500 ml was given at 20-30 minutes interval). Following the opening of the aorta the aortic valve was excised. The dilated aortic root and ascending aorta were removed and the coronary ostia were dissected free. Following this, the appropriate No-React^® ^BioConduit size was anastomosed to the aortic annulus with a continuous or interrupted sutures, then the coronary buttons were attached to the graft and finally the distal end of the conduit was anastomosed to the distal ascending aorta. In some cases in which the distal aorta was aneurysmatic, a woven Dacron graft with a side branch for reinstating arterial flow was inserted first using circulatory arrest at 17°C (oesophagus temperature) without utilizing cerebral perfusion.

### Data Collection and Postoperative Follow-up

Clinical outcomes were investigated for a mean follow-up of 7.1 ± 0.3 years (range of 2.2-10.5 years) and reported following the AATS/STS/EACTS 2008 guidelines [17]. Patients' data were obtained from hospital records, telephone interview and mailed questionnaire. The occurrence of death was obtained by reviewing the data from the Office of National Statistics Registry and contacting relatives or their general practitioners. The study, as well as the use of patient's data for research purposes and publication, was approved by the local Ethics Committee and, because this was a retrospective analysis of a well established surgical procedure and the investigations were performed as part of the standard care, patient's consent was not required.

The echocardiographic findings were collected from the existing records and the aortic valve pathology was qualitatively graded according to American Society of Echocardiography guidelines.

### Statistics and Expression of Results

Discrete variables are presented as number and as percentage. Continuous variables are presented as mean ± standard deviation (SD) or interquartile range depending on the normality deviation of the underlying distribution. Mean survival was estimated using Kaplan-Meier method. Three events were considered for the analyses: total mortality, cardiac mortality, and valve conduit related mortality. To estimate mean survival free from cardiac related fatal events those patients who died from non-cardiac causes were censored at the time of death. To estimate mean survival free from valve-conduit related fatal events, those patients who died from other causes considered non related with valve-conduit complications were also censored at the time of death.

## Results

Table 2 shows the surgical data. Up to 1/3 of the patients were operated as urgent, emergent or as a salvage procedure. 80.6% of the patients had an aneurysm of the aortic root and ascending aorta, 13.4% presented with acute dissection type A and 6% infection. The most used No-React^® ^BioConduit graft sizes were the 25 and 27 mm diameter. Also up to 1/3 of the patients received an associated surgical procedure with 17 requiring coronary artery bypass grafting (CABG) with the left internal mammary artery (IMA) or saphenous vein grafts (SVGs). In those with vein grafts, the proximal end of the SVG was attached to the ascending aorta through an opening made in the No-React^® ^BioConduit graft. The operative times (cardiopulmonary bypass, aortic cross-clamp and circulatory arrest) are also shown in Table 2. None of the patients were anticoagulated with warfarin but they received aspirin (75 mg/day) for life.

**Table 2:** Surgical data and postoperative complications

	N valid	
*Operative priority*	67	

Elective		45 (67.2%)

Urgent		15 (22.4%)

Emergency		4 (6%)

Salvage		3 (4.5%)

*Number of operations*	67	

First		56 (83.6%)

Second		10 (14.9%)

Third		1 (1.5%)

*Type of aortic valve lesion*	67	

Stenotic		18 (26.9%)

Regurgitant		35 (52.2%)

Mixed		14 (20.9%)

*Explanted valve*	67	

Native		52 (77.6%)

Bioprosthesis		7 (10.4%)

Mechanical		7 (10.4%)

Homograf		1 (1.5%)

*Aortic valve pathology*	67	

Calcific degeneration		29 (43.3%)

Myxomatous degeneration		11 (16.4%)

Prosthetic valve failure		9 (13.4%)

Congenital		5 (7.5%)

Annuloaortic ectasia		3 (4.5%)

Dissection		3 (4.5%)

Infection		2 (3%)

Rheumatic		1 (1.5%)

Other degenerative		1 (1.5%)

Unknown		3 (4.5%)

*Pathology of the aorta*	67	

Aneurysm		54 (80.6%)

Dissection		9 (13.4%)

Infection		4 (6%)

*No-React^® ^BioConduit size*	67	

20		1 (1.5%)

21		5 (7.5%)

23		7 (10.4%)

25		23 (34.3%)

27		24 (35.8%)

29		4 (6%)

31		1 (1.5%)

Unknown		2 (3%)

*Other cardiac procedures*	67	

CABG		17 (24.4%)

Replacement/repair of other valves		3 (4.5%)

LV Aneurysmectomy		1 (1.5%)

Other procedures		5 (7.5%)

*CBP time (min); mean (SD)*	65	183 (86) Range: 96-554

*CPB time (P25, P50, P75)*		135; 155; 207

*Aortic XC time (min); mean (SD)*	65	130 (45) Range: 63-275

*Aortic XC time (min) (P25, P50, P75)*		99.5; 117; 146.5

*Circulatory arrest time; mean (SD)*	28	33.71 (17) Range: 4-69

*Circulatory arrest time (P25, P50, P75)*		24.2; 28.5; 43.7

*Postoperative complications*		

Atrial fibrillation	67	17 (25.4%)

Low cardiac ouput	67	11 (16.4%)

Renal complication	67	5 (7.5%)

Neurological	67	3 (4.5%)

Implantation of PPM	67	3 (4.5%)

Pulmonary	67	2 (3%)

Infective complications	67	2 (3%)

G.I. complications	67	2 (3%)

Re-sternotomy for bleeding	67	2 (3%)

Sternal resuturing	67	1 (1.5%)

Readmitted to ITU	67	1 (1.5%)

### Early results

Eight patients (11.9%) died within the 30-day postoperative period. All of these patients were at an advanced state of disease: 5 patients were in cardiogenic shock, 1 patient had active aortic endocarditis, and 2 patients had complicated type A dissection of the aorta. Although the case with endocarditis is considered valve-related, the patient was in septic shock at the time of surgery and died the same operative day.

As shown in Table 2 atrial fibrillation was the commonest postoperative complication followed by development of low cardiac output. Three patients suffered from cerebral ischemic attacks, all of them resolving without permanent neurological deficit within a week and only 2 patients were re-operated for surgical bleeding non-related to the valved conduit.

### Late results

**Figure 1:**
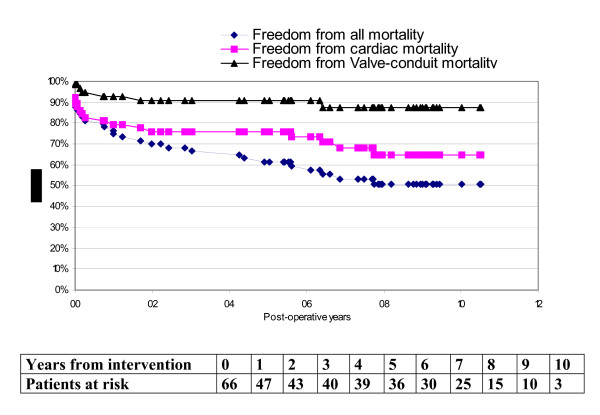
**Kaplan-Meier curves of mortality**.

Figure 1 shows that the overall 10-year survival following replacement of the aortic valve and root was 51% (mean survival time: 6.6 years; 95% CI 5.5-7.7). It also shows that the actuarial freedom from cardiac death was 65% at 10 years including operative mortality (mean survival free from cardiac related mortality: 7.6 years; 95% CI 6.5-8.7) whilst the actuarial freedom from device-related mortality was 88% for the same period (mean survival free from valve-conduit related mortality: 9.4 years; 95% CI 8.7-10.2), this including operative mortality.

**Figure 2:**
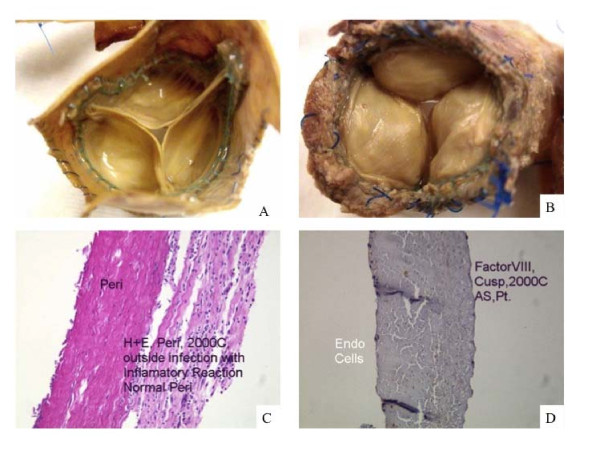
**Outflow and inflow of the explanted graft, as well as histological analysis of the valve. **Panel A shows the outflow of the valve, with totally normal looking, coapting cusps; Panel B shows the outflow, and the inflammatory reaction to the abscess which was responsible for valvular distortion is seen in the upper right hand corner; Panel C shows the H+E histological slide, showing normal looking pericardium of the conduit and the inflammatory cells of the abscess cavity; Panel D shows the Factor VIII immunoassay of one of the cusps, positive for monolayered endothelial cells.

After 7.1 ± 0.3 years (range of 2.2-10.5 years) follow-up, more than 50% of the survivors were in NYHA I/II and more than 60% of the survivors were angina-free (CCS 0). During this period, no thromboembollic events were recorded neither structural deterioration of the valved conduit. However, there were two cases with mild aortic regurgitation (+1 regurgitation only). In one of them, fever was developed 9 months prior being admitted to hospital in severe cardiogenic shock with both stenosis and regurgitation and endocarditis was diagnosed. She was urgently taken to the OR where an abscess outside the conduit was compressing and distorting and causing both stenosis and regurgitation. Upon explantation the valve and the conduit looked normal. Figure 2 shows the outflow and inflow of the explanted graft, as well as histological analysis of the valve. The pericardium of the conduit and the valve were unaffected by the infection that was exclusively located to the outside of the graft. Interestingly, the valve cusps and the pericardium of the prosthesis stained positive for Factor VIII immunoassay indicating the covering of the graft by a monolayer of endothelial cells. The source (human versus porcine) of the endothelial cells on the graft was not investigated but it will be expected that they are coming from the same patient since the process of graft preparation eliminates any endothelium of porcine origin. This case was the only one reoperated in this series.

### Echocardiographic findings

The echocardiografic findings (Tables 3 and 4) at last follow-up (mean = 4.3 years) showed that, in the studied patients, the valve function did not deteriorate and the diameter of the conduit was not increased, with no evidence of stenosis, dilatation, calcification or thrombosis of the graft.

**Table 3:** Echocardiography data prior to surgery (mean = 120 days; median = 90 days) and early (mean = 43.1 days; median= 7 days) and late (mean=4.3 years;  median= 4.4 years) after surgery

	N valid	Mean (SD)	Range	P25, P50, P75
*Aortic root diameter (mm)*				

Preop	26	51 (14.3)	26-83	41; 50; 57

First echo post-op	18	34.6 (5.5)	25-49	31; 34; 38

Last echo post-op	15	33.6 (4.3)	25-40	30; 33.5; 37

*LVIDd (mm)*				

Preop	11	64 (11.7)	45-78	53; 62; 75

First echo post-op	17	52 (8)	40-69	46; 52; 58

Last echo post-op	16	49 (7.3)	36-64	43; 48; 53

*Aortic velocity (m/sg)*				

Preop	10	2.6 (0.9)	1.4-4.7	1.9; 2.3; 3.1

First echo post-op	34	2.1 (0.5)	1-3.2	1.5; 2; 2.3

Last echo post-op	20	1.97 (0.6)	0.9-3.8	1.6; 1.9; 2

*Aortic max gradient*				

Preop	25	51 (31)	8.1-122	22; 50; 72

First echo post-op	38	17 (9.3)	4.3-42	10.3; 15.5; 21.2

Last echo post-op	26	16.2 (11)	3.5-59	10.6; 14.3; 18.3

*Aortic mean gradient*				

Preop	16	23.7 (21)	4.4-77	8; 17.5; 34.2

First echo post-op	37	9.5 (4.3)	3.3-23.3	5.8; 9; 12

Last echo post-op	22	9.4 (6.4)	3-34	6.1; 8; 9.4

*Aortic V2 VTI*				

Preop	8	51 (26)	27-110	35; 41; 59

First echo post-op	35	34.4 (11)	17-62	26; 32; 40

Last echo post-op	16	37 (16)	25-91	28.4; 31; 40.1

*LV ejection fraction*				

Preop	19			

> 50%		8 (12%)		

30-50%		8 (12%)		

< 30%		3 (4.5%)		

First echo post-op	15			

>50%		8 (12%)		

30-50%		2 (3%)		

< 30%		5 (7.5%)		

Last echo post-op	19			

> 50%		16 (24%)		

30-50%		2 (3%)		

< 30%		1 (1.5%)		

**Table 4:** Paired analysis for the first (mean = 43.1 days; median= 7 days) and  second (mean=4.3 years; median= 4.4 years) echocardiography investigations  performed after surgery

	N valid	Mean (SD)	Range	P25, P50, P75
*Aortic root diameter (mm)*	N = 7			

First echo post-op		33.4 (2)	30-36	32; 33.7; 35

Last echo post-op		33.3 (3.1)	29-38	30; 33.5; 3

*LVIDd (mm)*	N = 10			

First echo post-op		52 (10)	40-68	45.7; 46.4; 62

Last echo post-op		51 (7.7)	41-64	44; 49; 57

*Aortic velocity (m/sg)*	N = 13			

First echo post-op		2.1 (0.5)	1.4-3.2	1.6; 2.1;2.5

Last echo post-op		2.1 (0.7)	0.9-3.8	1.6; 2; 2.3

*Aortic max gradient*	N = 20			

First echo post-op		18 (10.4)	6-42	10; 15.5; 22.7

Last echo post-op		16 (12.2)	3.5-59	10.2; 14; 16

*Aortic mean gradient*	N = 18			

First echo post-op		9.2 (5.2)	3.3-23.3	5.5; 7.8; 11.2

Last echo post-op		9.7 (6.8)	3-34	6.7; 8; 9.3

*Aortic V2 VTI*	N = 13			

First echo post-op		32.7 (11.4)	21-62	24; 29; 39.8

Last echo post-op		38.6 (17)	25-91	28.5; 33; 43.9

Abbreviations: left ventricular internal diameter in diastole - LVIDd; aortic V2 VTI

## Discussion

The present study shows for the first time that the totally biological No-React^® ^BioConduit graft affords excellent long-term clinical results, with few graft-related complications, in a high risk group of patients (eg, elevated EuroScore) with short life expectancy. During the study period, no deterioration of the valve (eg, calcification, rupture) and conduit (eg, dilatation, calcification) were detected. These results are of clinical importance for the surgery of the aortic root and in some patients might represent a better alternative than other synthetic or biological valved conduits.

The 10-year results of aortic root replacement using composite grafts with mostly mechanical valves are less than desirable [2,5], mainly due to complications with anticoagulation and low, but consistent, rates of infection. There are several aortic valved conduits as alternative to a synthetic prosthesis with a mechanical aortic valve prosthesis but they also present their own set of specific issues that are discussed below. In biological aortic valved conduits, preservation of the graft collagen structure appears to be critical to avoid dilatation of the conduit and incompetence of the prosthetic valve. Pulmonary autografts have been shown to develop a high incidence of both dilatation of the conduit and incompetence of the valve [9-11] and they may not be suitable as substitute of the aortic root because their collagen structure and content differs form that of the aorta. In addition, in patients with cystic medial degeneration of the aorta, particularly those with bileaflet aortic valve and those with Marfan's syndrome, the pulmonary artery may also present with degenerative changes of the wall [18] that may make the autograft unsuitable for use as a substitute of the aortic root. Indeed, dilatation and regurgitation of the pulmonary autograft constitute the primary cause of failure and the principal reason for reoperation after the Ross procedure [9-11]. To overcome this problem, it has been proposed that in patients with bileaflet aortic valve the pulmonary autograft should be implanted with the use of the aortic root inclusion technique instead of aortic root replacement and that both the aortic annulus and the sinotubular junction should be fixed with a strip of Dacron fabric [19]. When an inclusion technique is not feasible, pulmonary autograft reinforcement with a Valsalva Gelweave Dacron tube (Terumo Cardiovascular Systems Inc, Ann Arbor, Mich) has been recommended as an option [20]. However, even with the use of the aortic root inclusion techniques, valve prolapse still remains the main cause of failure of the pulmonary autograft [21]. A systematic review of evidence on outcome after the Ross procedure has shown that, although the Ross procedure provides satisfactory results for both children and young adults, with outcomes also depending on the surgeon executing the procedure, durability limitations become apparent by the end of the first postoperative decade, in particular in younger patients [21]. It is worth mentioning that a broad spectrum of complex reoperations may be required after the Ross procedure which are associated with important morbididty [22].

The cause of the detrimental changes on pulmonary autografts are not fully elucidated but Schoof *et al*. [11] performing histological analysis on explanted specimens from the Ross procedure demonstrated that the elastic tissue of the autograft had been slowly substituted with fibrous material, including both the conduit wall and the valve cusps, a "degeneration" considered as a negative "remodelling" process. The observation of fibrous hyperplasia of the ventricularis and changes in the cellular and extracellular matrix characteristics in pulmonary autograft valve explants has lead to the suggestion that a primary valve-related cause is involved in pulmonary autograft valve failure [23].

Another alternative for the replacement of the aortic root is the use of allografts; however, cryopreserved aortic allografts are not easily available and also have an age-related limited durability. Thus, it has been shown that although the use of allografts for aortic valve replacement is associated with low occurrence rates of most valve-related events, over time the risk of structural valve deterioration increases, which is comparable to stented xenografts [24] and other aortic valve prostheses (Carpentier-Edwards pericardial and supra-annular valve, Medtronic Freestyle valve) [25]. Furthermore, Smedira et al. reported the explantation of 46 allografts after 5.6 ± 3.1 years follow-up in 744 patients whom have received cryopreserved allografts with a mean age of 49 ± 12 years. In this study, structural valve deterioration was the most frequent cause (59% of the cases) of valve-related reoperation after allograft aortic valve replacement [26]. Therefore there is a considerable lifetime risk of reoperation, especially in young patients, and, because of this, at some institutions the use of allografts only remains the preferred valve substitute for patients with active aortic root endocarditis and for patients in whom anticoagulation should be avoided.

The No-React^® ^BioConduit, being a completely biological and readily available graft, is an excellent alternative to pulmonary autografts and allografts. The BioConduit is easily handled facilitating its technically insertion. The absence of clinical evidence for degenerative changes of the No-React^® ^BioConduit graft seen in our study may be explained by the manufacturing process used. Thus, the recognition that glutaraldehyde and formaldehyde are prerequisites for limiting calcification and the importance of preservation of cross-linked collagen for the durability of biological tissue [27] was fundamental to develop the method used in the No-React^® ^BioConduit graft. In this process, heparin is used to lock the glutaraldehyde residue, so that glutaraldehyde leaching is abolished and its potential immunological reactivity is prevented [12], hence keeping all the advantages of glutaraldehyde but abolishing its side effects.

One important finding of our study was the rare occurrence of infection of the No-React^® ^BioConduit graft. One case with aortic endocarditis was in septic shock at the time of surgery dying the following day; therefore the lost of this patient cannot be attributed to infection of the newly implanted graft. The only other case presenting with late endocarditis was in fact a periprosthetic abscess without affecting the graft. Clinical studies with No-React^® ^valves, receiving an identical treatment to the No-React^® ^BioConduit, have also shown a high resistance to infection [14,15]. By contrast, synthetic aortic valved conduits [7], pulmonary autografts [28] and allografts [29] have an important rate of failure because of endocarditis. In the active phase of allografts with endocarditis the operative mortality and long-term prognosis are similar to those reported with conventional prostheses [30]. The reason for the resistance of No-React^® ^BioConduit and valve to infection is not fully understood but the presence of endothelium with No-React^® ^tissue on blood contacting surfaces has been suggested as a potential explanation [31]. Our results contrast with the recently reported degeneration of the No-React^® ^BioConduit in 7 of the 115 cases implanted with the prosthesis more than 1 year after surgery [16]. Endocarditis was identified as the most likely cause, although extensive microbiological examinations did not reveal a causative organism [16]. During the follow-up study period, we did not observed this complication in none of the patients implanted with this prosthesis. However, our study is up to 10 years and we believe it would be required at least 15 to 20 years to confirm whether the No-React^® ^BioConduit is really resistant to degeneration and infection, a question that probably should also be explored in a larger population in prospective and randomized studies comparing the No-React^® ^BioConduit with other biological conduits.

In conclusion, the present study has demonstrated that the No-React^® ^BioConduit does not dilate or deteriorate and resists infection after 10-year follow-up. Therefore, the No-React^® ^BioConduit may be a good alternative to other conduits for surgery of the aortic root in all age range.

## Competing interests

The authors declare that they have no competing interests.

## Authors' contributions

MG and AS performed the surgery, designed the study, analysed the results and participated in the writing of the manuscript. AM contributed to the collection of data. IF carried out the statistical analyses and also participated in the writing of the manuscript. All authors read and approved the final manuscript.
